# Pulmonary angiosarcoma in an HIV-positive patient presenting with hemoptysis and multisystem involvement: Report of a rare case

**DOI:** 10.3892/mi.2025.261

**Published:** 2025-08-19

**Authors:** Geran Maule, Saar Peles, Hisham Husham, Mohammad Khraisat, Abdallah Rayyan, Luis Javier

**Affiliations:** 1 College of Medicine, University of Central Florida, Orlando, FL 32827, USA; 2 HCA Florida North Florida Hospital, Graduate Medical Education Internal Medicine Residency Program, Gainesville, FL 32605, USA

**Keywords:** oncology, cardiology, angiosarcoma, immunology, multidiscipline

## Abstract

Pulmonary angiosarcoma is a rare, aggressive malignancy often mimicking other lung cancers. The present study describes the case of a 36-year-old male patient with human immunodeficiency virus (HIV) on highly active antiretroviral therapy who developed severe hemoptysis and respiratory distress, initially attributing his symptoms to mold exposure. Imaging revealed bilateral pulmonary nodules surrounded by ground-glass (suggestive of hemorrhagic lesions), and a biopsy confirmed stage IV pulmonary angiosarcoma. Immunohistochemical staining yielded positive results for ERG, CD31, CD34 and CD117, and echocardiography identified a large tricuspid valve mass, suggesting metastatic disease. He was commenced on paclitaxel treatment, but switched to doxorubicin following an infusion reaction. His course was complicated by recurrent hemothorax and pericardial effusions, requiring thoracentesis, chest tube placement and pericardiocentesis. Due to disease progression and frequent readmissions, he ultimately opted for hospice care. The case described herein illustrates the diagnostic complexity of pulmonary angiosarcoma in the setting of HIV and highlights the poor prognosis associated with extensive pulmonary and cardiac involvement.

## Introduction

Angiosarcoma is a rare and highly aggressive cancer originating from the endothelial cells of blood and lymphatic vessels, comprising <1% of sarcoma cases (1-3). This malignancy can affect any part of the body, but pulmonary involvement is particularly rare (4). Although the majority of cases occur spontaneously, risk factors such as radiation, chronic lymphedema, exposure to vinyl chloride and genetic syndromes have been documented in the literature (3). Primary pulmonary angiosarcoma is an aggressive but rare cancer of the pulmonary arteries that is malignant in nature. Secondary pulmonary angiosarcoma where lung metastasis from some other origin, such as the heart or breast occurs more often (4,5). Metastatic pulmonary angiosarcoma often presents with non-specific symptoms, complicating and delaying the diagnosis (4,6). Given the clinical and pathological similarities between primary and metastatic pulmonary angiosarcoma, excluding other tumor sites is often necessary for diagnosing primary pulmonary angiosarcoma (5). Angiosarcoma is the most common malignancy affecting the heart, and the lungs are the most common site of malignancy (7). Early detection plays a critical role in the workup and management of pulmonary angiosarcoma and having a high clinical suspicion augments early detection (4).

In pulmonary angiosarcoma, hemoptysis is a critical and alarming symptom indicative of tumor invasion into the pulmonary vasculature (8-11). Hemoptysis as an initial presentation is rare and is often accompanied by other symptoms related to other metastatic sites or systemic involvement (8-10). The non-specific presentation of primary angiosarcoma is even more complex in patients with other underlying comorbidities. In immunocompromised patients, hemoptysis can occur due to opportunistic infections (~80%), or other malignancies related to immunodeficiency, such as Kaposi's sarcoma (12,13). This overlapping symptomatology between pulmonary angiosarcoma, infections, and other malignancies can further delay diagnosis and appropriate treatment, further complicating the clinical picture. In terms of radiographic evidence, the review compiled by Yogi *et al* (14) indicated that the halo sign was observed in 58% of patients on a computed tomography (CT) scan of the chest. Among the patients of 28 cases of primary pulmonary angiosarcoma summarized in the study by Yogi *et al* (14), 50% of patients (14/28) had multiple nodules (14). Solitary lesions appeared in 39.3% of patients (11/28). They also noted that patients with multiple nodules had more aggressive courses and poorer prognosis compared to their single-lesioned counterparts (14).

The present study describes the case a case of metastatic pulmonary angiosarcoma in an human immunodeficiency virus (HIV)-positive patient presenting with hemoptysis and multisystem involvement. This serves to highlight the diagnostic challenges associated with this condition and the importance of considering rare malignancies in differential diagnoses, particularly in patients with complex medical histories.

## Case report

A 36-year-old African American male patient with HIV on highly active antiretroviral therapy who initially presented to the HCA Florida North Florida Hospital Emergency Department with 3 weeks of progressive hemoptysis (day 1). He reported recent exposure to mold following flooding and renovations in his apartment. His symptoms began as a mild, intermittent cough with clear sputum, which progressed to hemoptysis, shortness of breath, fatigue, post-tussive chest and back pain, and palpitations. Upon his arrival, he was alert but frail, with conjunctival pallor, borderline tachycardia and coarse breath sounds.

**Figure 1:**
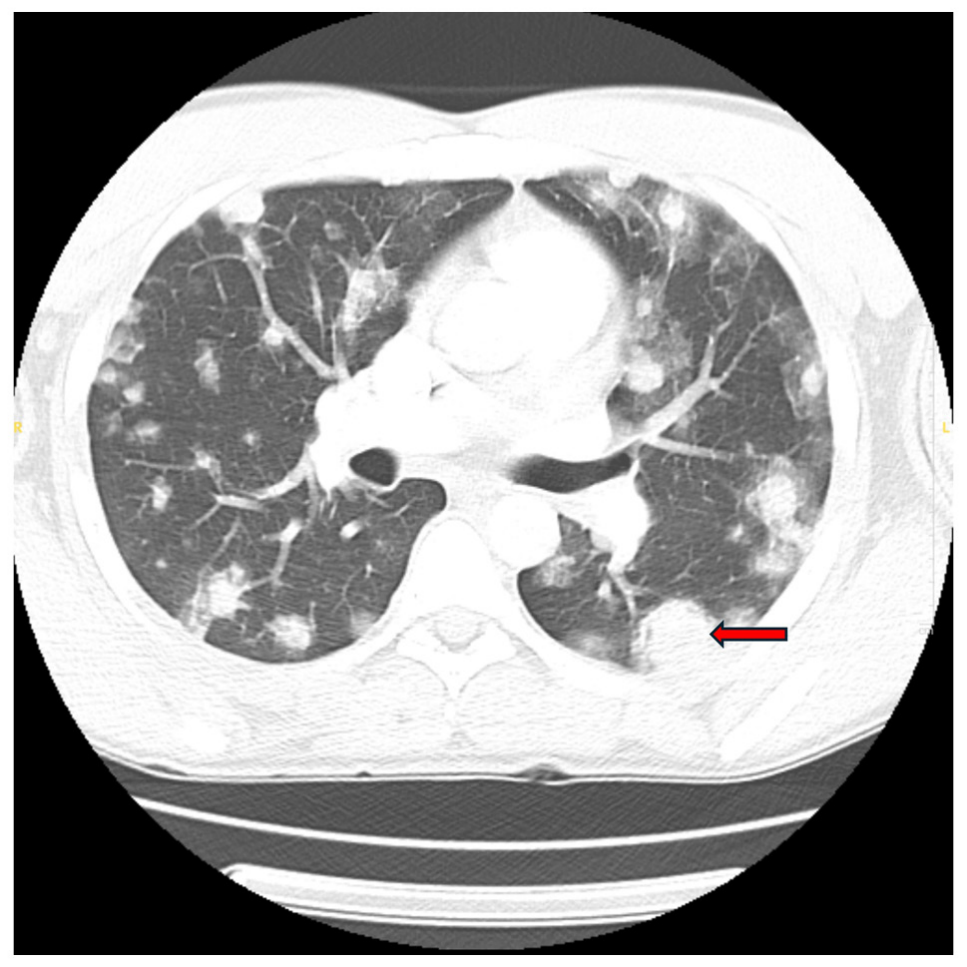
Chest computed tomography scan (day 1), illustrating bilateral pulmonary nodules with surrounding ground-glass halos, largest measuring 29x24 mm in the left lower lobe (red arrow).

Initial laboratory tests revealed microcytic anemia (Hgb, 8.4 g/dl), thrombocytopenia (platelets, 52 K/µl) and markedly elevated D-dimer levels (19,119 ng/ml). The CD4 count was 447, and the HIV viral load was undetectable. Imaging upon admission revealed innumerable bilateral pulmonary nodules with peripheral ground-glass halos, the largest measuring 29x24 mm in the left lower lobe, along with enlarged hilar lymph nodes (up to 14x15 mm on the left and 14x21 mm on the right), hepatic lesions and lytic bone lesions, all suggestive of metastatic disease (Fig. 1). Given his immunocompromised state, mold exposure history and imaging findings, initial concerns included opportunistic fungal infections, Kaposi's sarcoma and metastatic malignancy.

A robotic-assisted bronchoscopy with transbronchial biopsy and bronchoalveolar lavage (BAL) was performed on day 3. Blood was noted throughout the tracheobronchial tree, although no focal bleeding source was identified. The BAL fluid culture grew *Haemophilus influenzae*, and he was commenced on ceftriaxone 2 g daily for 5 days (day 6). Fungal, Acid-fast bacillus and tuberculosis workups were negative. Transthoracic echocardiography on day 8 revealed a large (7.4x3.3 cm) mobile mass on the atrial side of the tricuspid valve, initially raising concern for culture-negative endocarditis. In the setting of HIV, this prompted empiric treatment with continuation of ceftriaxone 2 g daily and vancomycin 1 g every 8 h, and serological testing for *Bartonella*, Q fever and *Brucella* was ordered. A dental evaluation was also pursued to assess for potential infectious sources. Blood cultures remained negative throughout his hospitalization periods.

**Figure 2:**
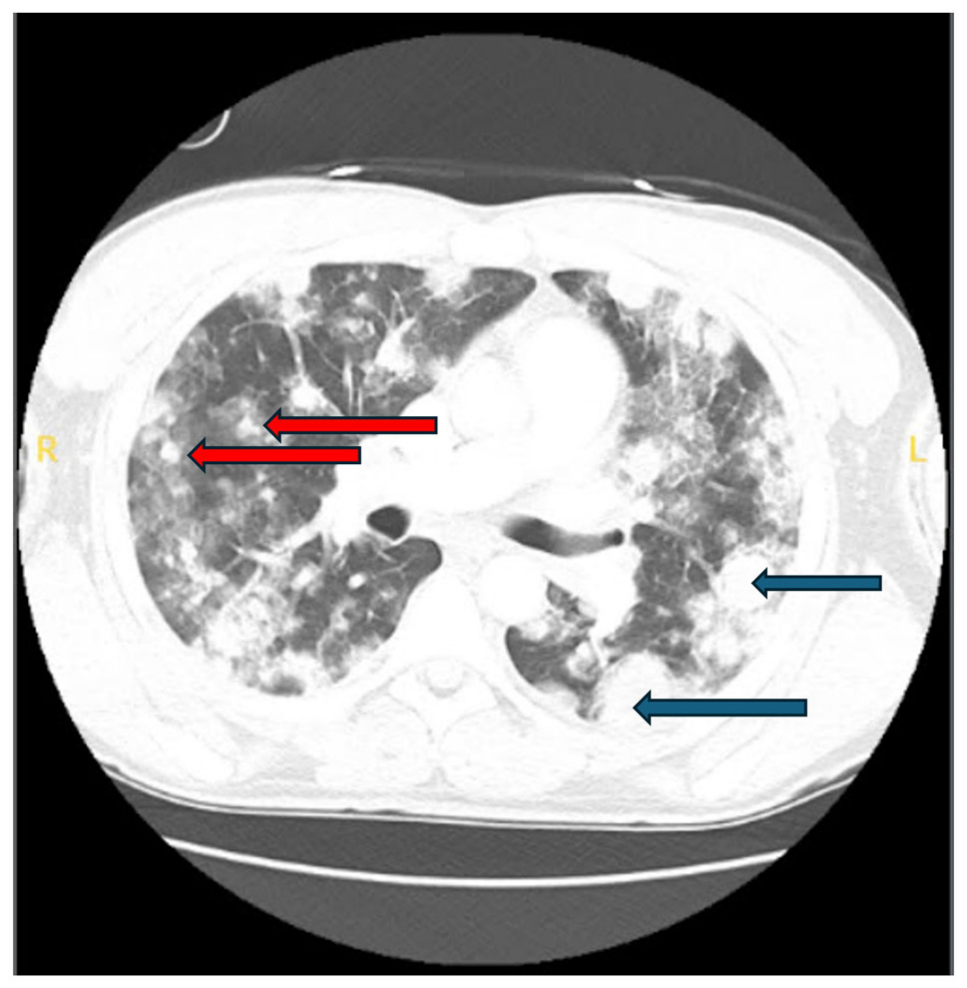
Chest computed tomography scan (day 14) illustrating the increased size and number of nodules. Bilateral pulmonary nodules are indicated by blue arrows, and pulmonary masses with surrounding ground-glass opacity are indicated by red arrows.

A CT scan on day 14 revealed an interval increase in the size and number of nodules, with the largest left-sided mass now measuring 4.2x3.1 cm, and a right-sided mass along the major fissure measuring 2.9x1.8 cm, along with new right hilar adenopathy measuring up to 2.3x1.6 cm (Fig. 2).

**Figure 3:**
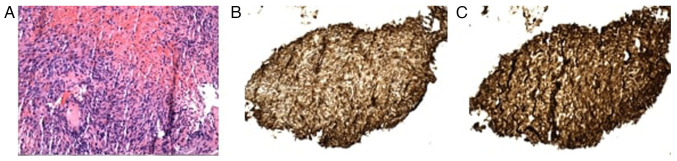
(A) H&E staining, x200 magnification illustrating poorly differentiated spindle cell neoplasm with moderate nuclear atypia, hemorrhage, and red blood cell extravasation; (B) CD34 stain, x200 magnification, positive; (C) CD31, x200 magnification, positive.

The patient departed from the hospital day 16, against medical advice (AMA), prior to the biopsy results, citing fatigue with the prolonged workup. He returned 5 days later (day 21) with worsening hemoptysis and dyspnea. Laboratory tests revealed a hemoglobin level of 6.6 g/dl and a platelet count of 37 K/µl. He was transfused and stabilized. The pathological analysis of the initial biopsy sample, reviewed at a tertiary center, confirmed stage IV pulmonary angiosarcoma with immunohistochemistry positivity for ERG, CD31, CD34 and CD117, and negativity for STAT6, HHV8 and ALK (Fig. 3). These results excluded more common HIV-associated malignancies, such as Kaposi's sarcoma and ruled out inflammatory myofibroblastic tumor and solitary fibrous tumor.

**Figure 4:**
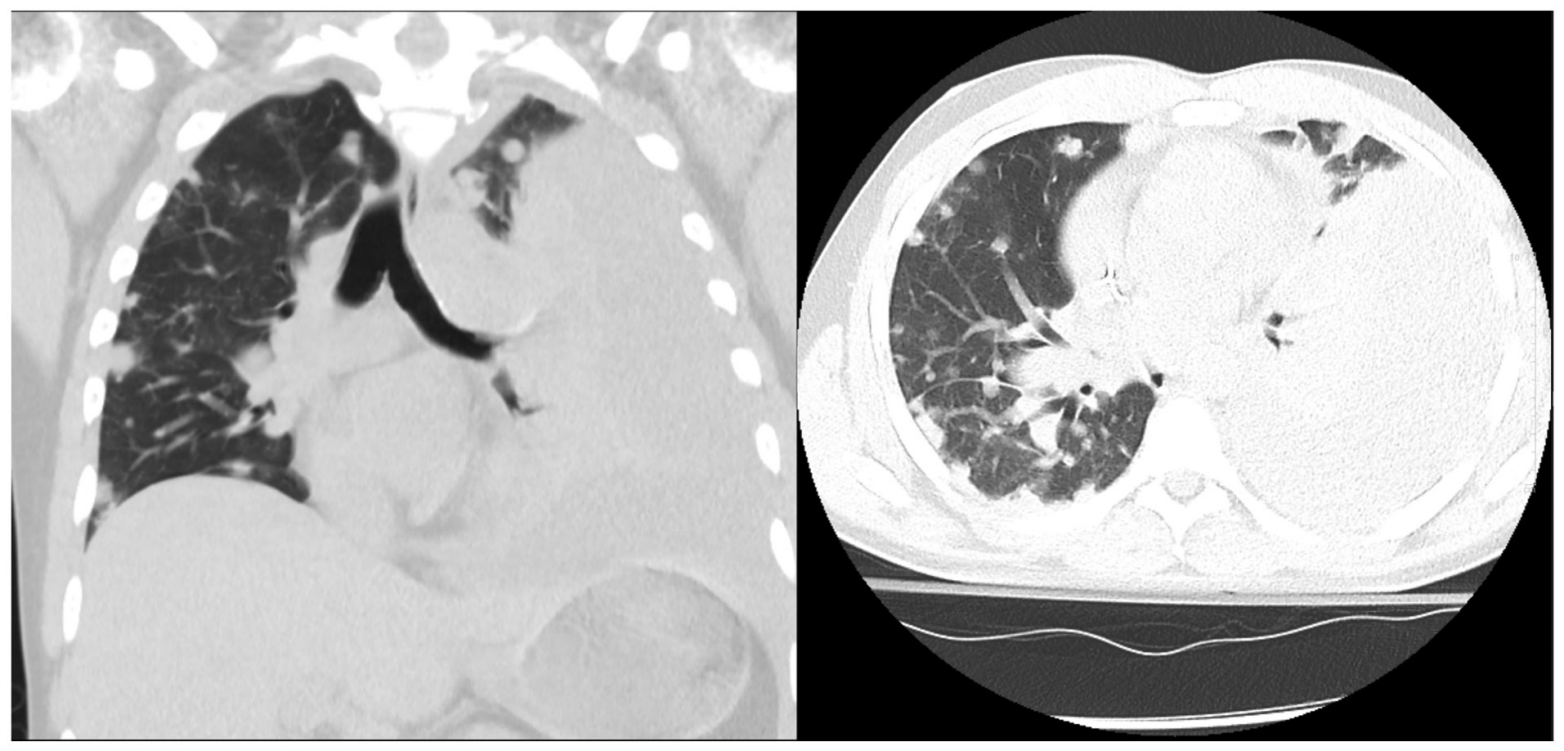
Coronal (left panel) and axial (right panel) non-contrast chest computed tomography scan (day 45) illustrating large left pleural effusion with near-complete lung collapse.

The Oncology Department initiated weekly paclitaxel therapy, 160 mg once weekly (3 weeks on, 1 week off) on day 24, a first-line agent with demonstrated efficacy in angiosarcoma. He initially tolerated the first dose, but again departed from the hospital, AMA, on day 29 before receiving subsequent treatment. He returned several days later (day 39) with severe dyspnea and was found to have a large left pleural effusion. Chest tube placement on day 45 drained 1.35 liters of bloody fluid, consistent with hemothorax, a common complication of angiosarcoma due to tumor-induced vascular fragility. Cytology from both the pleural and later pericardial effusions observed on the CT scan was negative for malignancy, with no malignant cells identified (Fig. 4).

**Figure 5:**
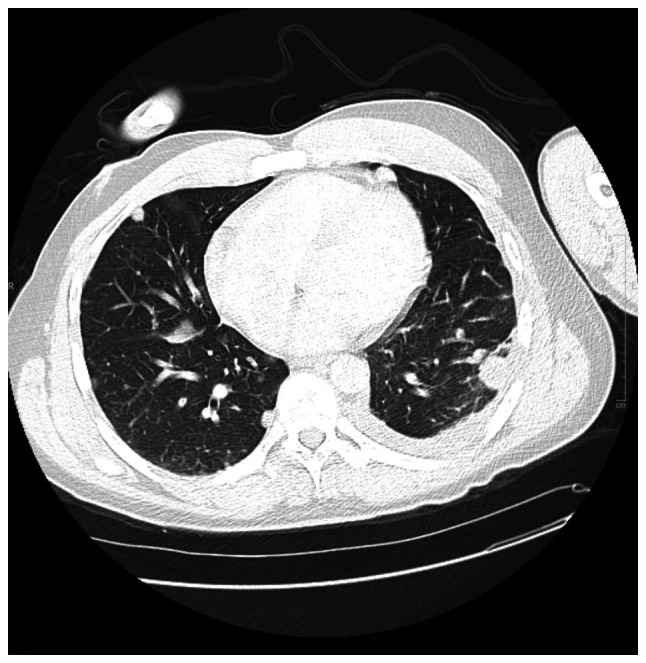
Non-contrast chest computed tomography scan (day 70) illustrating an improvement in the size and number of nodules following doxorubicin treatment.

During his second attempt at paclitaxel treatment on day 41, he experienced a grade 2 infusion reaction characterized by dyspnea and hypoxia, necessitating the discontinuation of treatment. Given the reaction severity and limited access to close outpatient monitoring, he was transitioned to doxorubicin 60 mg/m^2^. He received his first dose of doxorubicin on day 50 and tolerated it well. He continued with a second cycle on day 64 without major complications. Repeat CT imaging following two cycles of doxorubicin (day 70) demonstrated a marked improvement in pulmonary and hepatic metastases (Fig. 5), supporting a degree of chemotherapeutic responsiveness.

**Figure 6:**
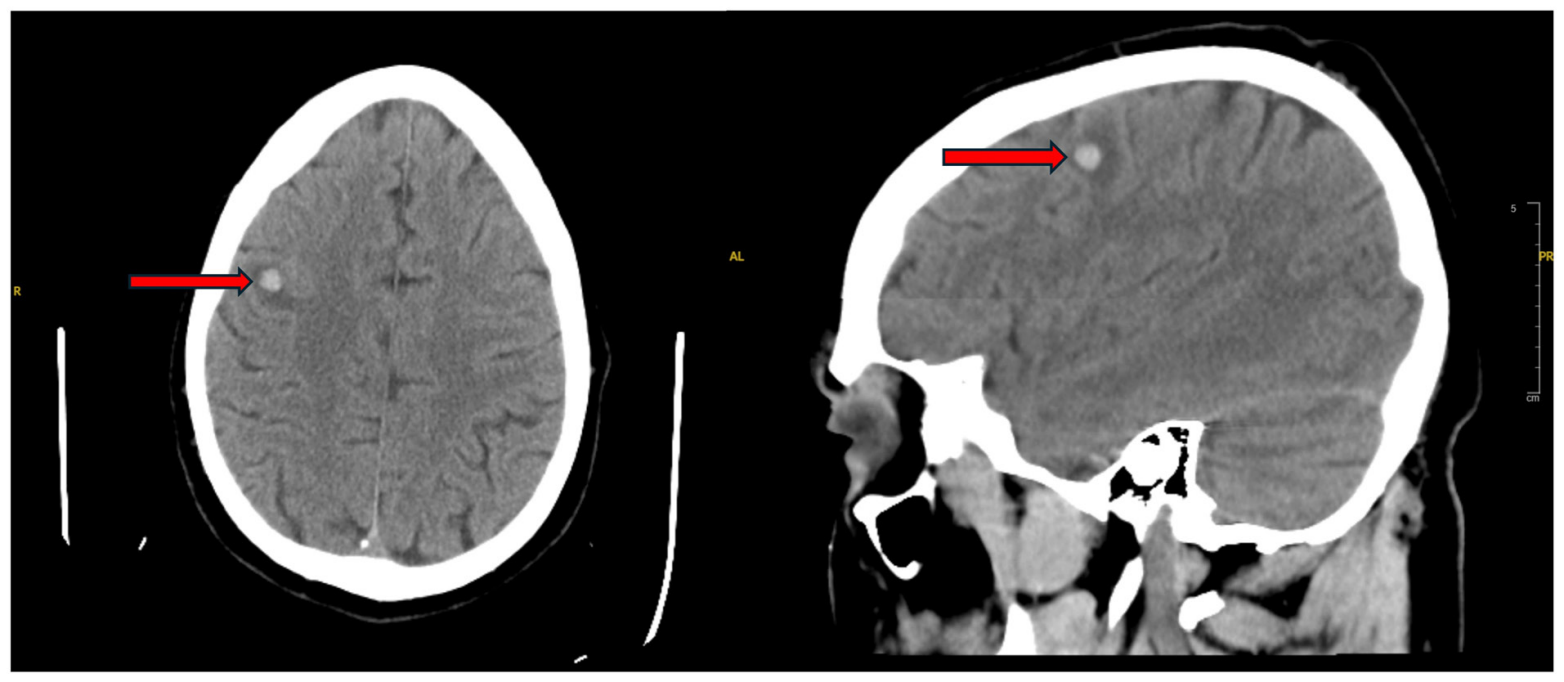
Axial (left panel) and sagittal (right panel) non-contrast head computed tomography scan (day 106) illustrating an 8-mm right frontal hemorrhagic metastasis with surrounding edema.

Despite early signs of treatment benefits, his course was complicated by a progressive pericardial effusion. On day 106 of hospitalization, shortly after using the bathroom, he experienced acute abdominal pain, diaphoresis, hypotension and oxygen desaturation. A rapid response was called, and a bedside echocardiogram revealed cardiac tamponade with right ventricular collapse. He was urgently transferred to the cardiovascular intensive care unit, where emergent pericardiocentesis was performed, draining 800 ml hemorrhagic fluid. He was stabilized with supportive care and was commenced on colchicine 0.6 mg twice daily, and ibuprofen 800 mg every 8 h for pericardial inflammation. Both medications were continued for 14 days while admitted, and he was discharged with the same regimen. A CT head scan performed during this admission revealed a right frontal hemorrhagic metastasis with surrounding edema (Fig. 6).

Although discharged home, he returned repeatedly over the following weeks with hemoptysis, shortness of breath and refractory metastatic pain. He was readmitted for fluid reaccumulation (day 85), requiring a second pericardiocentesis (day 90). Over time, his performance status declined. He initially enrolled in hospice care, but later revoked his do-not-resuscitate order (DNR) status and pursued further treatment following perceived clinical improvement. Despite this, the disease continued to progress, and he again decompensated. He and his family engaged in comprehensive goals-of-care discussions with the palliative care team. Expressing concern about suffering and being a burden to his loved ones, he opted for comfort-centered care and formally signed a DNR (day 109). He was discharged to hospice care on hospital day 135, where he later passed away peacefully. The clinical timeline is summarized in Table I.

## Discussion

Pulmonary angiosarcoma is a malignant, yet rare disease that has non-specific lung symptoms that lead to delay in diagnosis and attributability to more usual conditions (15-17). The case in the present study depicts the diagnostic and therapeutic challenges of the disease. The non-specificity of symptoms of hemoptysis, cough and dyspnea renders pulmonary angiosarcoma a clinical imitator that frequently imitates infectious pneumonia, tuberculosis and other malignancies (18,19). In immunocompromised patients, such as in patients with HIV, the initial suspicion tends to lean toward infectious disease rather than uncommon primary pulmonary malignancy.

Radiologically, pulmonary angiosarcoma tends to exhibit numerous pulmonary nodules in 50% of cases that possess characteristics of nodules, ground-glass opacities, or halo signs, a presentation that is typically associated with cases of fungal or hemorrhagic metastases (20,21). Tricuspid valve mass in this context was yet another clue for the differential diagnoses of cardiac angiosarcoma or metastatic disease. Primary pulmonary angiosarcoma could also be a possibility, as it has been shown to manifest as either single or multiple pulmonary nodules (22).

Histopathologic confirmation remained the gold standard, with strong positivity for CD31, ERG and CD34 supporting the diagnosis of angiosarcoma. These vascular markers are commonly expressed in endothelial malignancies and help distinguish angiosarcoma from other sarcomas and primary lung tumors. Negative staining for STAT6, ALK and HHV8 effectively excluded solitary fibrous tumors, inflammatory myofibroblastic tumors and Kaposi's sarcoma, respectively (23-27). CD117 (c-KIT), while also positive in this case, is less specific and can be expressed in a range of neoplasms, including gastrointestinal stromal tumors, seminomas and some melanomas. In angiosarcoma, CD117 expression has been reported but is considered supportive rather than definitive. Therefore, its diagnostic utility lies in being interpreted alongside more endothelial-specific markers like CD31 and ERG (28,29).

Pulmonary angiosarcoma is associated with a poor prognosis, particularly in patients with more than one pulmonary mass, who have much poorer outcomes than patients with a solitary mass (15). Accelerated disease course and poor responsiveness to chemotherapy account for poor survival in these patients (22). Paclitaxel remains a first-line chemotherapeutic agent with demonstrated efficacy in angiosarcoma; however, the patient described herein was unable to tolerate it, necessitating a switch to doxorubicin (22). While no standardized treatment regimen has been established, therapy selection is often guided more by patient tolerability than by effectiveness (22).

A unique characteristic of this case was the presence of a tricuspid valve mass that manifested either by direct invasion of the heart or by seeding from the primary tumor. Although primary cardiac angiosarcomas have their characteristic localization in the right atrium, secondary cardiac disease is generally more appreciated in the context of disseminated disease (30). This presentation provides an element of complexity to treatment as cardiac metastases have greater thrombotic potential and hemodynamic impairment (31). Hemorrhagic features of the effusions in angiosarcoma are in accordance with tumor-caused vascular fragility that leads to intrapulmonary and pericardial hemorrhage (32).

In conclusion, pulmonary angiosarcoma is an exceedingly rare and aggressive malignancy that presents significant diagnostic and therapeutic challenges, often mimicking infectious or other malignant processes. The present case report illustrates the diagnostic complexity of pulmonary angiosarcoma in the setting of HIV, where extensive pulmonary and cardiac involvement contributes to a poor prognosis, and highlights the importance of maintaining a high index of suspicion for angiosarcoma in immunocompromised patients presenting with unexplained pulmonary nodules, hemoptysis and cardiac abnormalities.

## Figures and Tables

**Table I:** Timeline of clinical events.

Time point	Clinical events
Day 1	Presentation with 3 weeks of hemoptysis. CT chest scan revealed bilateral nodules with halo sign. Placed in isolation. Initial labs show anemia, thrombocytopenia, high D-dimer levels.
Day 3	Robotic bronchoscopy with biopsy and BAL. Blood in airways noted. BAL sent for cytology and cultures.
Day 6	BAL culture grows *Haemophilus influenzae*. Ceftriaxone started. Fungal and TB workup negative.
Day 8	TTE revealed 7.4x3.3 cm mass on atrial side of tricuspid valve. Empiric vancomycin commenced for possible culture-negative endocarditis.
Day 13	Biopsy suggestive of spindle cell sarcoma. Sent for external review.
Day 16	Patient left AMA.
Day 21	Returned with worsening hemoptysis. Hgb 6.6, platelets, 37 K. Transfused. Biopsy confirmed angiosarcoma (ERG^+^, CD31^+^, CD34^+^, CD117^+^).
Day 24-25	Paclitaxel commenced. Port placed.
Day 29	Left AMA again, missed outpatient oncology follow-up.
Day 39	Returned with dyspnea. Imaging stable. Planned to resume chemotherapy inpatient.
Day 41	Grade 2 reaction to second dose of paclitaxel. Transitioned to doxorubicin.
Day 45	Became more hypoxemic. CT scan revealed large pleural effusion. Chest tube placed, drains 1.35 liters bloody fluid.
Day 54	Second cycle of doxorubicin administered. Imaging revealed a partial response.
Day 61	Discharged home.
Day 69	Returned with shoulder pain and dyspnea. CT scan revealed ~50% reduction in tumor burden. Scheduled for outpatient chemotherapy, discharged with pain medications.
Day 73	Returned for pain control due to a lack of outpatient medications, given another prescription and instructed to follow-up outpatient.
Day 85	Admitted with intractable nausea. Echocardiogram revealed moderate pericardial effusion.
Day 90	Pericardiocentesis performed; 800 ml hemorrhagic fluid removed.
Day 94	Discharged on colchicine and NSAIDs.
Day 106	Returned with another pericardial effusion and tamponade physiology. Emergent pericardiocentesis performed. Head CT scan revealed hemorrhagic brain metastasis.
Day 109	Signs DNR. Discharged to hospice.
Day 131	Returned with hemoptysis. Interventional pulmonology deemed bleeding non-localizable. Referred to hospice again.
Day 135	Returned once more with chest pain and hemoptysis. No further interventions pursued. Final discharge to hospice.

CT, computed tomography; BAL, bronchoalveolar lavage; TB, tuberculosis; TTE, transthoracic echocardiogram; AMA, against medical advice; NSAIDs, non-steroidal anti-inflammatory drugs; DNR, do-not-resuscitate.

## Data Availability

The data generated in the present study may be requested from the corresponding author.
